# Exploring the association between socioeconomic status and cardiopulmonary exercise testing measures: A cohort study based on routinely collected data

**DOI:** 10.1371/journal.pone.0328056

**Published:** 2025-08-12

**Authors:** Donna Shrestha, Nicholas A. Wisely, Theodoros M. Bampouras, Daren A. Subar, Cliff Shelton, Christopher J. Gaffney

**Affiliations:** 1 Lancaster Medical School, Lancaster University, Lancaster, United Kingdom; 2 Blackburn Research Innovation Development Group in General Surgery (BRIDGES), Blackburn, United Kingdom; 3 Department of Anaesthesia, Wythenshawe Hospital, Manchester, United Kingdom; 4 Manchester Academic Health Science Centre, University of Manchester, Manchester, United Kingdom; 5 School of Sport and Exercise Sciences, Liverpool John Moores University, Liverpool, United Kingdom; 6 Department of General Surgery, East Lancashire Hospitals NHS Trust, Blackburn, United Kingdom; Hamad Bin Khalifa University, QATAR

## Abstract

**Background:**

Cardiopulmonary exercise testing (CPET) provides objective measures of cardiorespiratory fitness and can support surgical risk stratification. As socioeconomic status is a factor known to influence patient health and outcomes, we analysed how CPET-derived measures vary across levels of socioeconomic status in patients being considered for elective surgery.

**Methods:**

A database of patients who underwent CPET between 2011 and 2024 was analysed. Measures including oxygen consumption (V̇O₂) at gas exchange threshold (GET), peak V̇O₂, and ventilatory equivalent for carbon dioxide (VE/V̇CO₂) were compared across socioeconomic deprivation quintiles. Multivariable linear and logistic regression models assessed the effects of age, sex, body mass index (BMI), Revised Cardiac Risk Index (RCRI), and deprivation quintiles on CPET measures. Hierarchical regression models incorporating the Indices of Deprivation (IoD) domains and Access to Healthy Assets and Hazards (AHAH) scores determined whether wider social determinants of health explained the variance in CPET measures.

**Results:**

A total of 3344 patients (2476 male) were included, referred prior to procedures in vascular (2006), colorectal (650), upper GI (267), urology (205), and other (216) surgical specialties. Lower socioeconomic status was associated with younger age (p < 0.001), higher BMI (p = 0.022), higher smoking prevalence (p < 0.001), and RCRI ≥3 (p = 0.013). CPET measures were lower in the most deprived quintile (Q1) compared to the least (Q5): mean GET was 11.0 vs. 11.5 ml·kg^-1^·min^-1^ and peak V̇O_2_ was 14.8 vs. 16.3 ml·kg^-1^·min^-1^ (p < 0.05). Deprivation remained an independent predictor of lower GET and peak V̇O_2_, even after adjustment. Several IoD and AHAH domains explained small but significant variance in CPET measures.

**Conclusion:**

Patients from more deprived areas exhibit risk factors for poor health and lower cardiorespiratory fitness as measured by CPET. These findings add to our understanding of socioeconomic disparities in physiological reserve among surgical patients and may support the need for more holistic approaches to peri-operative care.

## Introduction

A cardiopulmonary exercise test (CPET) provides objective measures of cardiorespiratory and metabolic fitness and is commonly used to aid the preoperative assessment of patients undergoing surgery. CPETs are increasingly being performed in the United Kingdom (UK), with around 30,000 tests performed annually [1]. The results are used to inform the prediction of a patient’s risk of postoperative complications, tailor patient optimisation and prehabilitation, and plan postoperative care [2,3]. CPET data can also aid patient-centred shared decision making, contributing to a more informed consent.

The COVID-19 pandemic shed light on the growing health disparities in the UK [4] and catalysed initiatives to reduce health inequalities, building on those set out in the 2019 National Health Service (NHS) Long Term Plan [5]. The NHS in England emphasised the need to restore services inclusively, analyse health inequalities using robust datasets, and accelerate preventative programmes targeting those at greatest risk of poor outcomes [6]. The Health and Care Act 2022 mandates that integrated care systems consider health inequalities in all decision making [7]. Regarding perioperative pathways, the 2023/24 NHS standard contract set out that providers must “implement a system of early screening, risk assessment and health optimisation for all adult service users waiting for inpatient surgery” [8]. Therefore, understanding how CPET can be applied in the context of significant health inequalities is an important consideration.

Socioeconomic deprivation is associated with poorer surgical outcomes, including higher rates of postoperative complications and mortality [9–11]. The reasons for these disparities are likely multifactorial and may stem from disparities in access and utilisation of healthcare services [12,13], lifestyle factors [14] and co-morbidities [15]. Patients from deprived backgrounds also report inferior healthcare experiences [16]. Identifying whether cardiorespiratory fitness differs by socioeconomic status could help uncover one potential physiological mechanism contributing to these inequalities.

Although CPET measures are known to predict adverse surgical outcomes [2], the relationship between socioeconomic deprivation and cardiorespiratory fitness in preoperative patients is yet to be explored. However, studies in healthy young adults do report lower levels of cardiorespiratory fitness in those from poorer socioeconomic backgrounds [17,18] and a meta-analysis including 9435 people from four population-level studies demonstrated a positive association between higher educational attainment and cardiorespiratory function [19]. These studies suggest that socioeconomic status is associated with differences in cardiorespiratory fitness in the general population, but it remains unclear whether similar patterns are observed in preoperative CPET measures among surgical patients.

Within the study setting of Greater Manchester, forty percent of patients who live within the areas served by the Greater Manchester Integrated Care Board live in the twenty percent most socioeconomically-deprived areas in England [20]. The region faces significant deprivation-related social challenges [21] and disparities in health outcomes such as life expectancy between small geographical areas [22].

By examining the relationship between socioeconomic factors and CPET measures, this study aims to inform equitable preoperative care strategies and contribute to efforts to reduce health inequalities. We hypothesised that patients from more socioeconomically deprived areas would have lower Gas Exchange Threshold (GET) and peak oxygen consumption (peak V̇O₂) values, and higher ventilatory equivalent for carbon dioxide (VE/V̇CO₂), even after adjusting for clinical and demographic factors.

## Methods

### Ethics

Ethical approval was granted by Lancaster University (FHM-2024–4326-IRAS-2) and the NHS Health Research Authority (24/HRA/1302). Data were collected prospectively as part of routine care, between 02/09/2011 and 19/04/2024. The requirement for individual patient consent was waived in accordance NHS data governance policies. Following ethical approval, the database was accessed for research purposes on 17/06/2024 by a member of the direct care team and an anonymised database was passed on to the researchers for analysis.

### Inclusion/Exclusion criteria

The patient cohort included individuals being considered for surgery, who were referred from a range of NHS sites in Greater Manchester. The major specialties from which they were referred included vascular, colorectal, urology, and upper gastrointestinal surgery, and all specialties were included in the study. Patients who were unable to cycle or did not have a postcode were excluded from the analysis.

### Study design

This study is a retrospective analysis of a prospectively collected database consisting of consecutive adult patients undergoing CPET at Wythenshawe Hospital, Manchester, UK.

Variables recorded consisted of patient characteristics including age, sex, body mass index (BMI), smoking status, revised coronary risk index (RCRI), static spirometry and CPET measures. The CPET measures that were included in the study were GET, peak V̇O2, and VE/V̇CO_2_ at GET. Of the spirometry measures, the forced expiratory volume in first second (FEV_1_) to forced vital capacity (FVC) ratio (FEV_1_/FVC) was used for the analysis.

Although commonly referred to as the “anaerobic threshold” in the clinical and perioperative literature [3], ‘GET’ is used in this study to reflect the physiological basis of the measurement, which corresponds to the first ventilatory threshold. A GET < 11 ml·kg^-1^·min^-1^ was used as an exploratory marker of reduced cardiorespiratory fitness, consistent with values cited in the perioperative literature as associated with poor outcomes of intra-abdominal surgery [23,24]. While optimal thresholds in GET for risk prediction may vary between surgical specialties [2], this value serves as a pragmatic reference point for comparison in preoperative assessment.

In the absence of a co-morbidity score or list, the RCRI was used as a surrogate for cardiovascular co-morbidities and FEV_1_/FVC for respiratory co-morbidities. An RCRI ≥3 is thought to be associated with a 15% (95% CI 11.1–20.0%) risk of major cardiac event, defined as death, myocardial infarction, or cardiac arrest at 30 days after noncardiac surgery [25] and this risk was compared by deprivation quintiles.

Analysis was performed on the whole dataset including all specialties and a sub-group analysis was carried out on vascular patients only (supplement 1).

### CPET methodology

Each CPET was undertaken by a Perioperative Exercise Testing and Training society (POETTS) accredited consultant anaesthetist and conducted in accordance with their consensus guidelines [3]. Tests were performed using an electromagnetically braked cycle ergometer (Corival CPET Cycle Ergometer, Lode B.V. Zernikepark 16 9747 AN Groningen, The Netherlands). The exercise protocol included a patient specific ramp of either 12, 15 or 20 Watts per minute aiming for around 10 minutes of exercise until symptoms limited. A rapid gas analyser (Ultima™ CardiO2® Gas Exchange Analysis System, MGC Diagnostics Corporation, 350 Oak Grove Parkway, Saint Paul, MN 55127) was used alongside continuous 12-lead ECG, pulse oxygen saturation and non-invasive blood pressure monitor, whilst the patient was at rest, undertaking unloaded cycling and pedalling against a predetermined and increasing resistance. Cardiorespiratory data was managed using Breeze Suite software (MCG Diagnostics, Saint Paul, MN, US). The GET was determined using a combination of the V-slope, ventilatory equivalents and ventilation curve analysis. Peak oxygen consumption was calculated by recording the highest value for oxygen consumption during the test [26].

### Index of multiple deprivation (IMD) quintiles

Patients’ English Index of Multiple Deprivation (IMD) 2019 deciles were derived from individual postcodes, to stratify the cohort by their neighbourhood deprivation as a surrogate for their socioeconomic status. The IMD incorporates scores from seven indices of deprivation (IoD) domains: ‘income’, ‘employment’, ‘health and disability’, ‘education, skills and training’, ‘crime’, ‘barriers to housing and services’, and ‘living environment’ [27]. These domains included a total of 37 indicators, each measuring the proportion of the population experiencing specific types of deprivation. In England, 32,844 Lower-layer Super Output Areas (LSOA), each containing an average of 1,500 residents, are ranked according to their IMD score. For this analysis, patients’ IMD deciles were re-categorised into quintiles, where Q1 represents the 20% most deprived areas, and Q5 the 20% least deprived areas.

### Access to healthy assets and hazards (AHAH) scores

In addition to the IoDs, the Access to Healthy Assets and Hazards (AHAH) database was also used to explore wider social factors which could contribute towards the variance in CPET measures. The AHAH index is an open access index of accessibility to, both positive and negative, environmental health-related amenities and exposures across LSOAs in Great Britain [28,29]. The AHAH domains consists of retail environment (access to fast food outlets, pubs, tobacconists, gambling outlets), health services (access to GPs, hospitals, pharmacies, dentists, leisure services), physical environment (blue space, green space – passive), and air quality (NO₂, PM10, SO₂). Version 3, released in 2022 was used for this analysis. In the case of IoD and AHAH domain scores, the higher the score, the greater the deprivation.

### Statistical analysis

Normality was confirmed if the ratio of skewness and kurtosis values to their respective errors was within ± 2. Baseline patient characteristics, CPET and spirometry measures were compared across the IMD quintiles (Q1-Q5) using descriptive statistics. For continuous variables, significant differences across the quintiles were assessed using one-way analysis of variance (ANOVA), followed by Tukey’s post-hoc test to determine inter-quintile differences and the corrected values are reported. For categorical variables, comparisons were made using log-linear regression.

Multivariable linear regression models were conducted to assess the association between each CPET outcome variable – peak V̇O2, GET and VE/V̇CO_2_ – and predictor variables including deprivation quintile, age, sex, BMI, smoking status, RCRI, and FEV_1_/FVC. Regression coefficients with 95% confidence intervals were reported to quantify the effect size of each predictor on the outcomes. A binomial multivariable logistic regression model was used to identify significant predictors of having GET < 11 ml·kg-1·min-1 and, specifically, whether deprivation was a significant predictor of GET < 11 ml·kg-1·min-1 when accounting for the other predictor variables. Odds ratios with 95% confidence intervals were reported to estimate the strength of associations.

Hierarchical multiple linear regression analyses were used to understand the association between IoD domain scores and AHAH domain scores, and three CPET outcome variables (peak V̇O_2_, GET and VE/V̇CO_2_). A baseline model, including age and sex, was generated to which each additional deprivation domain was added to create new models. Age and sex were included as baseline covariates in the first block of the hierarchical regression model as they are non-modifiable demographic factors known to influence CPET measures [30,31]. This approach allowed us to assess the additional variance in CPET outcomes explained by area-level deprivation domains (IoD and AHAH) beyond underlying physiological variation.

R^2^ values were reported, representing the overall model’s explanation of variance in each dependent variable. The change in R-squared (ΔR^2^) and F-statistic (F (ΔR^2^)) was reporting, representing the model’s ability to explain additional variance in the outcome variable with the addition of individual deprivation domains, whilst accounting for age and sex (included in the baseline model).

For all regression models, variables were checked for multicollinearity (variance inflation factor <5, tolerance >0.5) and Cook’s Distance values were within acceptable ranges (−2.5 to 2.5), confirming that no data transformation was required. Statistical significance was set at p < 0.05 for all analyses. Data analysis was conducted using Jamovi (version 2.4.8, The Jamovi Project, Sydney, Australia).

## Results

A total of 3344 patients underwent CPET between September 2011 and April 2024. Most patients were assessed for vascular surgery (2006, 60%), followed by colorectal 650 (19.4%), urology 205 (6.1%), upper GI 267 (8%), and other specialties accounted for 216 (6.5%).

Patient characteristics are reported in Table 1. The percentage of patients in each quintile, Q1 (most deprived) - Q5 (least deprived), was 28.7%, 18.7%, 15%, 18.8% and 18.8% respectively. 2476 (74%) patients were male and the proportion of males to females varied significantly across the deprivation groups. The mean patient age was 72.1 (9.6) years. There was a significant difference in age (p < 0.001) across the five deprivation groups; the mean age in the most deprived quintile (Q1) was 69.5 compared to 74.1 in the least deprived quintile (Q5). BMI was also higher in the most deprived quintile (Q1) (p = 0.022) with mean BMI 28.3 (6.4) vs. 27.5 kg.m^2^ (5.0) in Q1 and Q5, respectively.

**Table 1:** Comparison of patient characteristics by IMD quintile.

	Missing data	Q1	Q2	Q3	Q4	Q5	p-value **
**People in Greater Manchester Integrated Care Board (2023) (%)**		40.2	21.4	13.5	13.6	11.3	
**Total n (%)**		960 (28.7)	626 (18.7)	500 (15)	629 (18.8)	629 (18.8)	
**Age**	1	69.5,10.0	71.1, 9.9	72.9, 9.5	74.1, 8.5	74.1, 9.0	**<0.001**
Comparison to Q1*			**0.011**	**<0.001**	**<0.001**	**<0.001**	
Comparison to Q2*				**0.014**	**<0.001**	**<0.001**	
Comparison to Q3*					0.177	0.179	
Comparison to Q4*						1.000	
**BMI kg/m²**	15	28.3, 6.4	27.8, 5.3	27.6, 5.3	27.7, 5.4	27.5, 5.0	**0.022**
Comparison to Q1*			0.386	0.113	0.210	**0.022**	
Comparison to Q2*				00.958	0.998	0.794	
Comparison to Q3*					0.995	0.996	
Comparison to Q4*						0.928	
**Sex – Male n (%)**	0	684 (71.3)	460 (73.5)	367 (73.4)	482 (76.6)	483 (76.7)	**<0.001**
Comparison to Q1			0.333	0.385	**0.018**	**0.015**	
Comparison to Q2				0.975	0.198	0.176	
Comparison to Q3					0.212	0.190	
Comparison to Q4						0.947	
**Smoker n (%)**	1	308 (32.1)	148 (26.3)	111 (22.2)	90 (14.3)	84 (13.4)	**<0.001**
Comparison to Q1			**<0.001**	**<0.001**	**<0.001**	**<0.001**	
Comparison to Q2				0.568	**<0.001**	**<0.001**	
Comparison to Q3					**<0.001**	**<0.001**	
Comparison to Q4						0.616	
**Never smoked n (%)**	1	142 (14.8)	107 (17.1)	119 (23.8)	158 (25.1)	190 (30.2)	**<0.001**
Comparison to Q1			0.219	**<0.001**	**<0.001**	**<0.001**	
Comparison to Q2				**0.005**	**<0.001**	**0.001**	
Comparison to Q3					0.598	**0.017**	
Comparison to Q4						**0.046**	
**RCRI **≥** 3 n (%)**	1	127 (13.2)	57 (9.1)	40 (8)	69 (11)	56 (8.9)	**<0.001**
Comparison to Q1			**0.013**	**0.003**	0.181	**0.009**	
Comparison to Q2				0.512	0.272	0.900	
Comparison to Q3					0.095	0.589	
Comparison to Q4						0.221	
**FEV**_**1**_ **/FVC %**	27	65.9, 13.2	66.4, 13.2	70.2, 12.3	70.5, 12.3	70.0, 12.7	**<0.001**
Comparison to Q1*			0.954	0.165	**0.004**	**0.002**	
Comparison to Q2*				0.594	0.075	**0.042**	
Comparison to Q3*					0.868	0.766	
Comparison to Q4*						0.999	
**FEV**_**1**_ **/FVC < 70%** **n (%)**	27	513 (54.0)	339 (54.4)	244 (49.2)	294 (46.9)	310 (49.9)	**<0.001**
Comparison to Q1			0.872	0.083	**0.006**	0.113	
Comparison to Q2				0.083	**0.008**	0.113	
Comparison to Q3					0.443	0.809	
Comparison to Q4						0.284	

Patient characteristics (n = 3344), displayed for each IMD quintile (Q1 – most deprived to Q5 – least deprived). Data is presented as mean, SD, unless otherwise stated. Percentages are representative of the proportion of patients in each IMD quintile with the variable, apart from the first row which is a percentage of the total population. Significant p-values(p < 0.05) are in bold. *Tukey-adjusted post-hoc p-values. **Main effect of ANOVA.

There was a significant difference in smokers across the quintiles (p < 0.001), with a greater proportion of smokers in more deprived groups (Q1, 32.1% vs. Q5, 13.4%; p < 0.001) and, similarly, there was a significantly greater proportion of patients who have never smoked in the least deprived quintile (Q1, 14.8% vs. Q5, 30.2%; p < 0.001). There was a difference in the proportion of patients with RCRI ≥ 3 (main effect: p < 0.001; Q1, 13.2% vs. Q5, 8.9%; p = 0.013). The mean FEV_1_/FVC was significantly lower 65.9% (13.2) in Q1 compared to 70.0% (12.7) in Q5 (p = 0.002). The main differences in the proportion of patients with FEV_1_/FVC < 70% was seen in Q4 vs Q1 (46.9% vs. 54.0%, p = 0.006) and Q4 vs Q2 (46.9% vs. 54.4%, p = 0.008).

In comparison of CPET measures by IMD quintile (Table 2), GET varied significantly across the five quintiles (p = 0.001), with a difference of 0.5 ml·kg^-1^·min^-1^between Q1 and Q5 (p = 0.04). 9.2% more patients had a GET < 11 ml·kg-1·min-1 in Q1 than in Q5 (p = 0.002). Peak V̇O_2_ decreased with deprivation, with a difference in means of 1.5 ml·kg^-1^·min^-1^ between Q1 and Q5. VE/V̇CO_2_ varied significantly between Q1 and Q5 with a difference in means of 1.3, Q1-Q5 (35.9 ± 7.3 vs. 34.6 ± 6.6, p = 0.003). There was no significant difference in right or left hand grip strengths in the 1201 patients who had this measured.

**Table 2:** Comparison of CPET variables by IMD quintile.

	Missing data	Q1	Q2	Q3	Q4	Q5	p-value **
**Total n (%)**		960 (28.7)	626 (18.7)	500 (15)	629 (18.8)	629 (18.8)	
**GET (ml·kg** ^**-1**^ **·min** ^**-1**^ **)**	748	11.0, 2.2	11.3, 2.2	11.5, 2.6	11.3, 2.4	11.5, 2.5	**0.001**
Comparison to Q1*			0.123	**0.003**	**0.087**	**0.004**	
Comparison to Q2*				0.705	1.000	0.847	
Comparison to Q3*					0.759	0.998	
Comparison to Q4*						0.890	
**GET < 11 ml·kg** ^**-1**^ **·min** ^**-1**^ **n (%)**	748	346 (50%)	206 (42.2)	171(42.4)	232 (45.8)	207 (40.8)	**<0.001**
Comparison to Q1			**0.008**	**0.015**	0.140	**0.002**	
Comparison to Q2				0.948	0.260	0.658	
Comparison to Q3					0.316	0.626	
Comparison to Q4						0.113	
**peak V** **˙** **O**_**2 **_ **(ml·kg** ^**-1**^ **·min** ^**-1**^ **)**	39	14.8, 3.9	15.4, 3.8	16.1, 4.1	16.0, 4.1	16.3, 4.19	**<0.001**
Comparison to Q1*			**0.031**	**<0.001**	**<0.001**	**<0.001**	
Comparison to Q2*				0.061	0.072	**0.002**	
Comparison to Q3*					0.999	0.903	
Comparison to Q4*						0.767	
**VE/V** **˙** **CO**_**2**_	37	35.9, 7.3	35.1, 6.6	35.0, 6.6	35.1,6.3	34.6, 6.6	**0.006**
Comparison to Q1*			0.180	0.152	0.187	**0.003**	
Comparison to Q2*				1.000	1.000	0.712	
Comparison to Q3*					0.999	0.866	
Comparison to Q4*						0.699	
**Right Hand Grip (kg)**	2143	29.8,10.0	29.7,10.4	29.8,10.4	30.1, 9.9	29.8, 10.1	0.996
**Left Hand Grip (kg)**	2143	27.7, 9.6	27.7, 9.9	27.5, 9.7	28.6, 9.7	27.6, 9.58	0.750

CPET variables comparison displayed for each IMD quintile (Q1 – most deprived to Q5 – least deprived). Data is presented as mean, SD, unless otherwise stated. Percentages are representative of the proportion of patients in each IMD quintile with the variable, apart from the first row which is a percentage of the total population. Significant p-values(p < 0.05) are in bold. *Tukey-adjusted post-hoc p-values. **Main effect of ANOVA.

The logistic regression model, for GET < 11 ml·kg^-1^·min^-1^ (Table 3), was statistically significant, χ^2^(13) = 435, p < 0.001. The model explained 20.9% (Nagelkerke R^2^) of the variance in GET < 11 ml·kg ⁻ ¹·min ⁻ ¹ and correctly classified 72.8% of cases. Age, sex, BMI, RCRI and FEV_1_/FVC were significant predictors of having a GET < 11 ml·kg^-1^·min^-1^. Female sex increased the likelihood of having a GET < 11 ml·kg^-1^·min^-1^ significantly (OR 3.97 [CI 3.20, 4.92], p < 0.001). Adjusting for patient characteristics, compared to Q1, patients in Q2, 3, and 5 were less likely to have a GET < 11 ml·kg^-1^·min^-1^ (OR 0.73, p = 0.016; 0.74, p = 0.034; 0.72, p = 0.013, respectively). Smoking was not a significant factor in predicting a GET < 11 ml·kg^-1^·min^-1^ (p > 0.05).

**Table 3:** Results of regression models.

	GET < 11 ml·kg^-1^·min^-1^		GET ml·kg^-1^·min^-1^		peak V̇O2 ml·kg^-1^·min^-1^		VE/V̇CO_2_		
Predictor	OR [CI]	p-value	Estimate (β)	p-value	Estimate (β)	p-value		Estimate (β)	p-value
Age	1.04 [1.03, 1.05]	**<0.001**	−0.04 [−0.05, −0.03]	**<0.001**	−0.10 [−0.11, −0.09]	**<0.001**		0.17 [0.15, 0.19	**<0.001**
Sex (F-M)	3.97 [3.20, 4.92]	**<0.001**	−1.59 [−1.79, −1.39]	**<0.001**	−2.97 [−3.24, −2.69]	**<0.001**		0.89 [0.41, 1.36]	**<0.001**
BMI	1.10 [1.08, 1.12]	**<0.001**	−0.11 [−0.13, −0.10]	**<0.001**	−0.20 [−0.22, −0.17]	**<0.001**		−0.24 [−0.28, −0.20]	**<0.001**
Current Smoker (Yes-No)	0.98 [0.78, 1.23]	0.853	−0.06 [−0.28, 0.15]	0.572	−0.61 [−0.91, −0.30]	**<0.001**		1.61 [1.08, 2.14]	**<0.001**
RCRI (2−1)	1.85 [1.53, 2.23]	**<0.001**	−0.75 [−0.93, −0.56]	**<0.001**	−1.19 [−1.46, −0.92]	**<0.001**		1.04 [0.58, 1.51]	**<0.001**
RCRI (3−1)	3.78 [2.68, 5.34]	**<0.001**	−1.47 [−1.79, −1.14]	**<0.001**	−2.45 [−2.90, −2.00]	**<0.001**		2.43 [1.65, 3.22]	**<0.001**
RCRI (4−1)	8.78 [3.81, 20.21]	**<0.001**	−2.40 [−3.04, −1.76]	**<0.001**	−4.06 [−4.90, −3.22]	**<0.001**		4.13 [2.60, 5.66]	**<0.001**
RCRI (5−1)	3.37 [0.83, 13.75]	0.090	−1.93 [−3.24, −0.62]	**0.004**	−2.42 [−4.55, −0.29]	**0.026**		1.86 [−1.86, 5.57]	0.327
FEV_1_/FVC	0.98 [0.97, 0.99]	**<0.001**	0.03 [0.02, 0.03]	**<0.001**	0.06 [0.05, 0.07]	**<0.001**		−0.11 [−0.12, −0.09]	**<0.001**
IMD quintile (2−1)	0.73 [0.56, 0.94]	**0.016**	0.27 [0.03, 0.52]	**0.029**	0.40 [0.05, 0.75]	**0.027**		−0.78 [−1.39, −0.17]	**0.012**
IMD quintile (3−1)	0.74 [0.56, 0.98]	**0.034**	0.46 [0.19, 0.72]	**<0.001**	1.05 [0.67, 1.43]	**<0.001**		−1.07 [−1.74, −0.41]	**0.002**
IMD quintile (4−1)	0.85 [0.65, 1.09]	0.203	0.30 [0.05, 0.55]	**0.018**	1.07 [0.71, 1.43]	**<0.001**		−0.95 [−1.58, −0.33]	**0.003**
IMD quintile (5−1)	0.72 [0.56, 0.93]	**0.013**	0.34 [0.09, 0.59]	**0.007**	1.16 [0.80, 1.52]	**<0.001**		−1.43 [−2.06, −0.80]	**<0.001**

*Multivariable logistic and linear regression models with independent variables – GET < 11 ml·kg-1·min-1, GET, peak V̇O2, and VE/V̇CO*_*2*_*. Q1 (most deprived) is the reference value for deprivation. Significant p-values (p < 0.05) are in bold.*

The regression model, for GET (Table 3), was statistically significant F(13) = 51.6, p < 0.001. The model explained 20.4% (adjusted R^2^ = 0.204) of the variance in GET. Age, female sex, BMI, and RCRI were significant negative predictors of GET, whilst FEV_1_/FVC was a significant positive predictor of GET. Compared to Q1, Q2-Q5 had a significant positive association with GET, (Q5 vs Q1, β = 0.34, p = 0.007). Smoking was not a significant independent predictor for GET.

The regression model, for peak V̇O_2_ (Table 3), was statistically significant F(13) = 99.6, p < 0.001. The model explained 28.2% (adjusted R^2^ = 0.282) of the variance in peak V̇O_2_. Age, female sex, BMI, smoking and RCRI had a significant negative association with peak V̇O_2_. Deprivation was a significant negative predictor of peak V̇O_2_, with a β value of 1.16 [CI 0.80, 1.52] for Q5 vs Q1 (p < 0.001), suggesting that individuals in the least deprived quintile had a peak V̇O_2_ that was 1.16 ml·kg^-1^·min^-1^ greater than the most deprived quintile.

The regression model, for VE/V̇CO_2_ (Table 3), was statistically significant F(13) = 72.0, p < 0.001. The model explained 22% (adjusted R^2^ = 0.220) of the variance in VE/V̇CO_2_. Age, female sex, smoking, and RCRI were significant predictors of higher (adverse) VE/V̇CO_2_. Conversely, BMI, FEV_1_/FVC and all levels of deprivation were associated with lower (more favourable) VE/V̇CO_2_. However, patients in the least deprived quintile (Q5) had a VE/V̇CO_2_ that was 1.43 lower than the most deprived quintile, Q1 (β = −1.43 [CI −2.06, −0.80], p = 0.001).

In the hierarchical regression model, to explain the variance in GET (Table 4), the baseline model, including age and sex were significant predictors (F = 106.2, p < 0.001), and explained 7.6% (R^2^ = 0.076) of the variance in GET. Of the IoD domains, four of seven indicators, including the ‘health deprivation and disability score’, ‘employment score’, ‘education, skills and training score’, and ‘crime score’ each contributed a significant but small (1–1.6%) amount in explaining the variance in GET (p < 0.001). Of the five AHAH domain scores, air quality, passive green space, and retail domain scores explained between 0.1%−0.3% of further variance in GET after adjusting for age and sex.

**Table 4:** Association between GET and IoD and AHAH domains.

Predictors	R²	F (R²)	p-value (R²)	ΔR²	F (ΔR²)	p-value (ΔR²)
Indices of deprivation (2019) domain scores						
Age, Sex (baseline model)	0.076	106.2	**<0.001**	–	–	–
+ Health deprivation and disability score	0.092	87.7	**<0.001**	0.016	46.9	**<0.001**
+ Employment score	0.088	83.4	**<0.001**	0.012	34.9	**<0.001**
+ Income deprivation score	0.087	82.7	**<0.001**	0.012	32.9	**<0.001**
+ Education, skills, and training score	0.087	82.3	**<0.001**	0.011	31.8	**<0.001**
+ Crime score	0.086	81.3	**<0.001**	0.010	29.3	**<0.001**
+ Barriers to housing and services score	0.077	71.5	**<0.001**	7.05x10^-4^	1.98	0.160
+ Living environment deprivation score	0.076	70.9	**<0.001**	1.07x10^-4^	0.301	0.583
Access to Healthy Assets & Hazards (AHAH) domain scores						
+ Air quality domain score	0.085	80.7	**<0.001**	0.001	27.4	**<0.001**
+ NVDI value indicating Passive Green Space	0.080	75.4	**<0.001**	0.005	12.70	**<0.001**
+ Retail domain score	0.079	73.8	**<0.001**	0.003	8.48	**0.004**
+ Health domain score	0.076	71.4	**<0.001**	5.71x10^-4^	1.60	0.206
+Distance to nearest leisure facility	0.076	70.8	**<0.001**	3.61x10^-6^	0.010	0.920

*Predictor variables are in descending order of association with GET. Significant p-values (p < 0.05) are in bold.*

In the hierarchical regression model, to explain the variance in peak V̇O_2_ (Table 5), the baseline model, including age and sex were significant predictors (F = 206, p < 0.001), and explained 11.1% (R^2^ = 0.111) of the variance in peak V̇O_2_. Of the IoD domains, five of seven predictors, including ‘health deprivation and disability score’, ‘employment score’, ‘income score’, ‘education, skills and training score’, and ‘living environment score’ were significant predictors of peak V̇O_2_. These predictors explained between 0.04–2.9% of the additional variance from the baseline model. All AHAH domain scores were predictive of peak V̇O_2_. The air quality score explained the greatest additional variance of 1.3%.

**Table 5:** Association between peak V̇O_2_ and IoD and AHAH domains.

Predictors	R²	F (R²)	p-value (R²)	ΔR²	F (ΔR²)	p-value (ΔR²)
Indices of deprivation (2019) domain scores						
Age, Sex (baseline model)	0.111	206	**<0.001**	–	–	–
+ Health deprivation and disability score	0.140	179	**<0.001**	0.029	111	**<0.001**
+ Employment score	0.138	175	**<0.001**	0.027	101	**<0.001**
+ Income deprivation score	0.138	176	**<0.001**	0.027	104	**<0.001**
+ Education, skills, and training score	0.136	173	**<0.001**	0.025	96	**<0.001**
+ Living environment deprivation score	0.115	143	**<0.001**	0.004	15.3	**<0.001**
+ Crime score	0.111	138	**<0.001**	2.09 x10^-4^	0.774	0.379
+ Barriers to housing and services score	0.111	138	**<0.001**	8.74 x10^-5^	0.324	0.569
Access to Healthy Assets & Hazards (AHAH) domain scores						
+ Air quality domain score	0.124	156	**<0.001**	0.013	49.0	**<0.001**
+ Retail domain score	0.119	148	**<0.001**	0.008	28.5	**<0.001**
+ NVDI value indicating Passive Green Space	0.119	148	**<0.001**	0.008	28.2	**<0.001**
+ Health domain score	0.116	144	**<0.001**	0.004	16.4	**<0.001**
+Distance to nearest leisure facility	0.112	139	**<0.001**	0.001	4.05	**0.044**

*Predictor variables are in descending order of association with peak V̇O*_*2*_*. Significant p-values(p < 0.05) are in bold.*

In the hierarchical regression model, to explain the variance in VE/V̇CO_2_ (Table 6), the baseline model, including age and sex were significant predictors were significant predictors (F = 135, p < 0.001) and explained 7.6% (R^2^ = 0.076) of the variance in VE/V̇CO_2_. ‘Income’, ‘employment, education, skills and training’, ‘health deprivation and disability’, and ‘crime’ scores were all significant predictors of VE/V̇CO_2_, accounting for 0.1–1.6% of variance in VE/V̇CO_2_. Of the AHAH domains, green space availability and retail domain scores were statistically significant predictors and explained 0.1–0.3% further variance in VE/V̇CO_2_.

**Table 6:** Association between VE/V̇CO_2_ and IoD and AHAH domains.

Predictors	R²	F (R²)	p-value (R²)	ΔR²	F (ΔR²)	p-value (ΔR²)
Indices of deprivation (2019) domain scores						
Age, Sex (baseline model)	0.076	135	**<0.001**	–	–	–
+ Income deprivation score	0.092	111	**<0.001**	0.016	57.3	**<0.001**
+ Employment score	0.091	110	**<0.001**	0.015	53.5	**<0.001**
+ Education, skills, and training score	0.090	109	**<0.001**	0.014	51.2	**<0.001**
+ Health deprivation and disability score	0.088	107	**<0.001**	0.013	45.4	**<0.001**
+ Crime score	0.077	92.0	**<0.001**	0.001	4.97	**0.026**
+ Barriers to housing and services score	0.076	90.6	**<0.001**	3.00 x10^-4^	1.07	0.300
+ Living environment deprivation score	0.076	90.6	**<0.001**	2.71 x10^-4^	0.970	0.325
Access to Healthy Assets & Hazards (AHAH) domain scores						
+ NVDI value indicating Passive Green Space	0.079	94.2	**<0.001**	0.003	11.2	**<0.001**
+ Retail domain score	0.077	91.6	**<0.001**	0.001	3.91	**0.048**
+ Health domain score	0.077	91.4	**<0.001**	9.65x10^-4^	3.45	0.063
+ Air quality domain score	0.076	91	**<0.001**	5.98 x10^-4^	2.14	0.144
+Distance to nearest leisure facility	0.076	90.4	**<0.001**	1.58x10^-4^	0.566	0.452

*Predictor variables are in descending order of association with VE/V̇CO*_*2*_*. Significant p-values(p < 0.05) are in bold.*

## Discussion

This study is the first in the UK to explore the relationship between social determinants of health and cardiorespiratory function, in adult patients who have undergone CPET as part of an elective surgical pathway. We report four key findings: 1) patients from more deprived areas had greater cardiovascular and respiratory risk factors, while they also had a lower age and higher BMI; 2) Deprivation was an independent risk factor for lower cardiorespiratory function; 3) a gradient of adverse risks with increasing deprivation was apparent in terms of BMI, smoking status, FEV_1_/FVC, and three CPET measures; 4) Several indicators for the wider determinants of health were small but significant factors in explaining the variance in CPET measures. We discuss these findings in turn and consider their implications for understanding socioeconomic disparities in cardiorespiratory fitness and how the findings may inform equitable perioperative care strategies.

1. **Age, BMI, and comorbidity differences by socioeconomic deprivation**

In our cohort, patients from more deprived quintiles were younger but had a higher BMI, greater prevalence of smoking, and more respiratory compromise, as indicated by lower FEV1/FVC ratios. They were also more likely to have a RCRI score ≥3 and at greater risk of multimorbidity, which is consistent with the Scottish database study of over 1.7 million of the general population by Barnett et al. [15]. In the emergency surgical setting, Poulton et al. demonstrated similar trends in deprivation-related age and co-morbidities in the National Emergency Laparotomy Audit data [32]. This clustering of cardiovascular and respiratory risk factors among younger, more deprived patients may suggest that physiological decline occurs earlier in this group and may partly explain the variation in CPET performance across socioeconomic deprivation quintiles.

1. **Association between CPET measures and socioeconomic deprivation**

In our cohort, patients from deprived areas were more likely to have a lower GET and a GET < 11 ml·kg^-1^·min^-1^_._ The GET represents the V̇O_2_ at which carbon dioxide production begins to rise disproportionately to oxygen uptake. While GET is considered less dependent on maximal effort than peak V̇O₂ and is widely used as a reproducible marker of functional capacity, its identification can vary depending on protocol, patient characteristics (such as age, sex and comorbidities), and early test termination [31,33]. In our study, 712 patients who had a peak V̇O_2_ recorded, did not have a GET documented, likely reflecting these factors. This finding is consistent with larger clinical cohort studies such as Marzolini et al [31], where an identifiable GET was only observed in 69% of females and 88% of males, despite structured protocols. In our data, missing GET was more common in the most deprived quintiles, Q1 (26.25%) and Q2 (21.25%) versus Q4 (18.76%) and Q5 (18.44%). This pattern may reflect differences in exercise tolerance or premature fatigue in more deprived patients, potentially linked to underlying comorbidities or reduced physiological reserve [34]. It also highlights the importance of a nuanced interpretation of CPET results within the broader clinical context, as incomplete tests may disproportionately affect those already at higher risk.

Patients in the most deprived groups also had a significantly lower peak V̇O_2_, i.e., the highest V̇O_2_ value achieved during the CPET. Quintile 1 had a mean peak V̇O_2_ of 14.8 ml·kg^-1^·min^-1^, which is clinically significant, as a peak V̇O_2_ < 15 ml·kg^-1^·min^-1^ is a significant predictor of early mortality in patients following AAA repair [35]. A lower peak V̇O_2_ in the more deprived groups could be, partly, reflective of greater risk of co-morbidities.

A VE/V̇CO_2_ > 34 has been shown to be predictive of adverse postoperative outcomes [2,36]. The mean VE/V̇CO_2_, in our study, ranged between 34.6–35.9, with increasing values from the most to least deprived quintiles.

1. **The social gradient in risk factors and CPET measures**

The ‘social gradient’ in health, as demonstrated across our findings, is a well-established phenomenon [37] described across a range of health risks and outcomes in the general population, including smoking [14], multimorbidity [15] and premature mortality [38]. Health inequalities impact across the socioeconomic spectrum and is not limited to those in the most deprived socioeconomic groups [37]. Greater psychosocial stressors [39] and reduced access to health services [40,41], further compound the effects of deprivation. Evidence also suggests that the cumulative effect of adverse risks is greater in patients from more deprived backgrounds and that they are disproportionately affected [42,43].

Another possible explanation for the observed social gradient in health involves the biosocial perspective [44–46], which considers the interplay between exogenous socio-environmental factors and endogenous biological processes. It offers a holistic view of the individual, their susceptibility to disease, ability to adapt to adverse risks from a physiological, psychological and social perspective, and goes beyond attributing differences to “traditional” patient modifiable risk factors. Although our study did not measure biological markers, emerging evidence suggests that socioeconomic status, for instance, is associated with differences in gut microbiome independent of healthy diet, BMI and health deficits [47], potentially affecting inflammation and metabolic function [48]. Similarly, variation in epigenetic markers, linked with accelerated ageing [49], inflammation and responses to stress [50,51] have been demonstrated in patients from different socioeconomic backgrounds. These biological shifts, amongst others, could contribute to the deprivation-related variance in cardiorespiratory fitness through impaired metabolic efficiency [52,53]. While speculative, this hypothesis highlights the need for future research that integrates social and biological determinants of health and are important considerations as we strive to practice personalised medicine [54].

1. **The wider determinants of health and CPET measures**

From a wider public health perspective, we considered the variance in CPET measures in terms of a range of social determinants of health. Factors such as health deprivation, employment, income, education, living environment, air quality, green space availability, and access to adverse retail environments were all small but significant contributors to the variance in cardiorespiratory fitness and are critical areas for policymakers to consider in efforts to improve population health. An understanding of these broader determinants, and the ways they intersect to impact individuals, can guide the multidisciplinary team in designing prehabilitation programs. Providing support that is responsive to these factors requires a coordinated approach between primary care, secondary care, and local authorities, incorporating strategies like social prescribing, partnerships with community organisations, and initiatives to overcome individual logistical barriers. Future research should focus on understanding patients’ needs to inform service design that is equitably accessible and sensitive to the intersectionality of deprivation-related factors.

### Informing equitable perioperative care strategies

Although this study focuses on patients being considered for planned surgery, the deprivation-related differences in cardiorespiratory function we observed are reflective of those in non-surgical populations [19]. Cardiorespiratory fitness is modifiable and, in the general UK population, is an independent risk factor for all-cause mortality [55,56]. In our study, patients from more deprived backgrounds were younger and exhibited poorer cardiopulmonary fitness along with a higher prevalence of risk factors for adverse health. These findings highlight the importance of the NHS’ ‘making every contact count’ approach [57] which promotes early opportunistic interventions to improve health before patients reach the point of surgical referral or preoperative assessment. However, in the context of perioperative care, given our findings, and that patients from deprived backgrounds also face greater challenges in managing their health [40,58], an earlier and more comprehensive assessment could be beneficial.

The differences in cardiorespiratory fitness by socioeconomic status observed in our study have important implications for prehabilitation. In the surgical patient population, multimodal prehabilitation is potentially an effective intervention in modifying cardiorespiratory fitness. This has been demonstrated in randomised controlled trials through improvements in functional capacity, measured by increases in 6-minute walking test (6MWT) or CPET measures [59,60], corresponding to improvements in postoperative outcomes for both cancer and benign surgical patients [61,62]. Importantly, CPET measures can guide personalised goals and exercise interventions in prehabilitation [63], particularly when using CPET protocols that reliably enable identification of key metabolic thresholds such as the GET [33,64]. This represents a departure from a ‘one size fits all’ approach to exercise. Embedding this personalisation into routine perioperative care could help maximise benefit, particularly for patients with lower baseline fitness and higher preoperative risk, as seen in more deprived groups.

CPET, if used equitably, could provide vital information for patient optimisation. In addition to its role in patient postoperative risk stratification, CPET, along with spirometry, can be used to identify undiagnosed or unoptimised cardiorespiratory conditions, which could prompt further investigations [65]. For example, ECG changes in response to exercise, or delayed heart rate recovery after exercise correspond with RCRI≥3 and increased adverse cardiac events [66].

Health inequalities are evident throughout the perioperative pathway, with patients from deprived backgrounds experiencing longer waiting times for elective surgery [67]. Moving away from a ‘waiting list’ model to a ‘preparation list’ provides a unique opportunity to bridge this gap [68,69]. By using this time proactively through patient engagement initiatives, such as ‘surgical schools,’ healthcare professionals can enhance health literacy and provide culturally nuanced, holistic and inclusive support. For patients from deprived backgrounds, early identification of risk through comprehensive preoperative assessment, and tailored support during this period could help alleviate the adverse effects of deprivation. This is particularly important, as patients from socioeconomically disadvantaged backgrounds are often those who stand to benefit the most from prehabilitation but may be less likely to participate in such programs [70].

### Additional findings

In addition to the observed differences by socioeconomic status, our analysis also identified important disparities in CPET measures by sex, which may have further implications for personalised risk stratification and optimisation strategies. The differences in CPET measured by sex have been previously reported by Thomas et al. in 703 patients, who reported significantly lower GET and peak V̇O_2_ in females, after adjusting for weight [71]. Similar findings of differences in peak V̇O_2_ were reported in the post hoc analysis of the METS study, which additionally identified optimal sex-specific peak V̇O_2_ thresholds for postoperative complication prediction [30]. Our analysis adds to these findings; in our cohort, females were almost four times more likely to have a GET < 11 ml·kg^-1^·min^-1^, after adjusting for age, BMI, smoking, RCRI, FEV_1_/FVC, and deprivation. Similarly, adjusting for these, females were likely to have significantly lower GET by 1.59 ml·kg^-1^·min^-1^, peak V̇O_2_ by 2.97 ml·kg^-1^·min^-1^ and greater VE/V̇CO_2_ by 0.89. Other significant findings by Thomas et al. included that, on average, of the participants included in 17 studies related to perioperative CPET, 68.5% were male. In our cohort, 74% were male. This may be reflective of the epidemiology of the surgical specialties involved (e.g., vascular surgery), but more research is required to clarify whether this is the case. For example, although peripheral artery disease and abdominal aortic aneurysms are more prevalent in men [72], this would not account for our 80% male vascular cohort. Sex-related inequalities in the surgical pathway have been described in several areas [73–75] but the mechanisms remain under researched. Tailoring interventions to address these physiological differences could optimise outcomes for both sexes, especially in patients from higher-risk groups, though further research is needed to support this.

In our cohort, there were greater proportions of patients in the least deprived quintiles compared to the deprivation distribution within the Greater Manchester Integrated Care Board. Given that research suggests that deprived patients have a higher incidence of medical conditions and utilise greater NHS resources [76], we would expect the deprivation distribution in our cohort to reflect that of the general population or be skewed towards more deprived patients. Possible explanations for this discrepancy could include a greater tendency for emergency rather than elective presentations amongst more deprived groups, higher levels of self-advocacy in more affluent patients, or clinician bias in referrals for CPET. Further research into the patient pathway would be required to ensure equitable access at each stage. However, it should be noted that our cohort consisted of 60% vascular patients. For those being referred following abdominal aortic aneurysm screening, the referral pathway is clearly defined. The results are not dissimilar where a subgroup analysis was performed for vascular patients alone (supplement 1).

### Strengths

Despite the known challenges in identifying metabolic thresholds in clinical CPET settings, particularly in heterogeneous or deconditioned populations [77] this study has several methodological strengths. First, it draws on a large cohort spanning over a decade of routine CPETs. Second, all tests were conducted using cycle ergometry with ramp-incremental protocols, which are more conducive to threshold detection than treadmill-based step protocols. Third, the testing protocol was standardised with interpretation by POETTS-accredited consultant anaesthetists. Notably, approximately 60% of tests were performed by a single consultant anaesthetist, reducing inter-operator variability, with the remaining tests conducted by five other trained consultant anaesthetists.

### Limitations

This study is not without limitations. First, it should be acknowledged that, even when standardised ramp-incremental protocols are applied and tests are conducted by trained personnel, many CPET protocols remain suboptimal for the accurate determination of metabolic thresholds such as the GET. Recent evidence shows that a significant proportion of clinical CPETs fail to detect GET reliably, which may impact both the interpretation and the comparability of fitness-related variables [33].

Second, the Index of Multiple Deprivation (IMD), as an area-level socioeconomic measure, does not capture individual-level deprivation, which could introduce some misclassification of socioeconomic status at the individual level. Second, the RCRI and FEV_1_/FVC ratio were used as surrogates for comorbidities due to the unavailability of detailed comorbidity data. Additionally, there is heterogeneity within the study cohort, as it includes patients from multiple specialties and some who may not have proceeded to surgery. Not all patients who underwent CPET were subsequently placed on a surgical waiting list, which may limit comparability with studies that included only patients who ultimately underwent surgery.

Furthermore, the database did not capture each patient’s specific diagnosis. While surgical specialty and broad categories of intended procedures were available, these do not distinguish between underlying pathology or indicate the severity or the functional impact of the condition. Future prospective studies should aim to collect more granular clinical data to adjust for clinical factors that may influence CPET performance.

We also acknowledge that our study is based on data from a single healthcare trust and is representative of the specific socioeconomic and healthcare challenges locally, limiting the generalisability to other populations. A national CPET database, or future studies in multi-centre cohorts, would allow for external validation of our findings. Nevertheless, a strength of the study is that it offers insights that can be contextualised to the region, that might otherwise be obscured in national-level datasets.

## Conclusion

In summary, our study highlights significant socioeconomic disparities in cardiorespiratory fitness among preoperative patients, emphasising the need for equity-focused approaches in perioperative care. By incorporating both medical and social determinants into prehabilitation and risk stratification, healthcare providers can better support patients, ultimately contributing to improved surgical outcomes and addressing broader health inequalities.

## Supporting information

S1 File Vascular patient analysis. (DOCX)
