# Supplementary material for: Exploring the association between socioeconomic status and cardiopulmonary exercise testing measures: A cohort study based on routinely collected data
Source: PLoS One. 2025 Aug 12;20(8):e0328056. doi: 10.1371/journal.pone.0328056 (PMC12342313; doi:10.1371/journal.pone.0328056)
Supplement: S1 File — (DOCX) [file pone.0328056.s001.docx]

**Supplement 1 – Vascular patient analysis**

**Vascular patients only – baseline characteristics by IMD quintile.** Percentages displayed are as a total of each IMD quintile, apart from the first row.

|  | **Missing data** | **Q1 (most deprived)** | **Q2** | **Q3** | **Q4** | **Q5 (least deprived)** | **p-value**** |
| --- | --- | --- | --- | --- | --- | --- | --- |
| **Total n (%)** |  | 585/2006  (29.2%) | 395/2006  (19.7%) | 296/2006  (14.8%) | 388/2006  (12.9%) | 342/2006  (17.1%) |  |
| **Age (mean, SD)** | 1 | 70.9, 9.31 | 71.8, 9.29 | 73.7, 8.93 | 75.1, 7.68 | 75.3, 7.48 | **<0.001** |
| Comparison to Q1* |  |  | 0.415 | **<0.001** | **<0.001** | **<0.001** |  |
| Comparison to Q2* |  |  |  | 0.038 | **<0.001** | **<0.001** |  |
| Comparison to Q3* |  |  |  |  | 0.198 | 0.138 |  |
| Comparison to Q4* |  |  |  |  |  | 0.999 |  |
| **BMI (mean, SD)** | 5 | 27.3, 5.26 | 27.5, 4.77 | 27.1, 4.67 | 27.3, 4.84 | 27.4, 4.62 | 0.897 |
| Comparison to Q1* |  |  | 0.981 | 0.979 | 1.000 | 0.999 |  |
| Comparison to Q2* |  |  |  | 0.855 | 0.988 | 0.999 |  |
| Comparison to Q3* |  |  |  |  | 0.983 | 0.948 |  |
| Comparison to Q4* |  |  |  |  |  | 0.999 |  |
| **Sex – Male (n (%))** |  | 447/585  (76.4%) | 318/395  (80.5%) | 240/296  (81.1%) | 322/388  (83.0%) | 289/342  (84.5%) | **<0.001** |
| Comparison to Q1 |  |  | 0.129 | 0.115 | **0.014** | **0.003** |  |
| Comparison to Q2 |  |  |  | 0.850 | 0.369 | 0.156 |  |
| Comparison to Q3 |  |  |  |  | 0.518 | 0.253 |  |
| Comparison to Q4 |  |  |  |  |  | 0.581 |  |
| **Smoker (n (%))** | 1 | 220/585  (37.6%) | 110/395  (27.9%) | 81/296  (27.4%) | 72/387  (18.6%) | 63/342  (18.4%) | **<0.001** |
| Comparison to Q1 |  |  | **0.002** | **0.003** | **<0.001** | **<0.001** |  |
| Comparison to Q2 |  |  |  | 0.888 | **0.002** | **0.003** |  |
| Comparison to Q3 |  |  |  |  | **0.007** | **0.007** |  |
| Comparison to Q4 |  |  |  |  |  | 0.949 |  |
| **Never smoked (n (%))** | 1 | 43/585  (7.4%) | 35/385  (9.1%) | 34/296  (11.5%) | 63/387  (16.3%) | 67/342  (19.6%) | **<0.001** |
| Comparison to Q1 |  |  | 0.392 | **0.042** | **<0.001** | **<0.001** |  |
| Comparison to Q2 |  |  |  | 0.256 | 0.002 | **<0.001** |  |
| Comparison to Q3 |  |  |  |  | 0.077 | **0.006** |  |
| Comparison to Q4 |  |  |  |  |  | 0.244 |  |
| **RCRI**≥**3** |  | 76/585  (13.0%) | 30/395  (7.6%) | 24/296  (8.1%) | 43/388  (11.1%) | 32/342  (9.4%) | **<0.001** |
| Comparison to Q1 |  |  | **0.008** | **0.032** | 0.374 | **0.097** |  |
| Comparison to Q2 |  |  |  | 0.804 | 0.095 | 0.391 |  |
| Comparison to Q3 |  |  |  |  | 0.196 | 0.579 |  |
| Comparison to Q4 |  |  |  |  |  | 0.444 |  |
| **FEV_1_/FVC**  **(mean, SD)** | 27 | 64.4, 13.2 | 64.2, 13.1 | 65.2, 12.2 | 66.6, 13.0 | 66.4, 13.0 | **0.017** |
| Comparison to Q1* |  |  | 0.997 | 0.931 | 0.081 | 0.178 |  |
| Comparison to Q2* |  |  |  | 0.837 | 0.061 | 0.132 |  |
| Comparison to Q3* |  |  |  |  | 0.611 | 0.765 |  |
| Comparison to Q4* |  |  |  |  |  | 0.999 |  |
| **FEV_1_/FVC < 70%** | 27 | 343/580  (59.1%) | 243/392  (62.0%) | 168/295  (56.9%) | 206/386  (53.4%) | 190/338  (56.2%) | **<0.001** |
| Comparison to Q1 |  |  | 0.373 | 0.535 | 0.076 | 0.386 |  |
| Comparison to Q2 |  |  |  | 0.182 | **0.015** | 0.113 |  |
| Comparison to Q3 |  |  |  |  | 0.352 | 0.852 |  |
| Comparison to Q4 |  |  |  |  |  | 0.443 |  |

*Patient characteristics (n = 2006), displayed for each IMD quintile (Q1 – most deprived to Q5 – least deprived). Data is presented as mean, SD, unless otherwise stated. Percentages are representative of the proportion of patients in each IMD quintile with the variable, apart from the first row which is a percentage of the total population. Significant p-values(p<0.05) are in bold. *Tukey-adjusted post-hoc p-values. **Main effect of ANOVA.*

|  | **Missing data** | **Q1 (most deprived)** | **Q2** | **Q3** | **Q4** | **Q5 (least deprived)** | **p-value**** |
| --- | --- | --- | --- | --- | --- | --- | --- |
| **Total n (%)** |  | 585/2006  (29.2%) | 395/2006  (19.7%) | 296/2006  (14.8%) | 388/2006  (12.9%) | 342/2006  (17.1%) |  |
| **GET ml·kg^-1^·min^-1^ (mean, SD)** | 500 | 11.1, 2.15 | 11.4, 2.04 | 11.7,2.56 | 11.4,2.22 | 11.5,2.23 | **0.007** |
| Comparison to Q1* |  |  | 0.395 | **0.006** | 0.290 | **0.046** |  |
| Comparison to Q2* |  |  |  | 0.467 | 1.00 | 0.869 |  |
| Comparison to Q3* |  |  |  |  | 0.553 | 0.956 |  |
| Comparison to Q4* |  |  |  |  |  | 0.923 |  |
| **GET <11 ml·kg^-1^·min^-1^** | 500 | 188/399  (47.1%) | 126/294  (42.9%) | 97/236  (41.1%) | 131/306  (42.8%) | 107/271  (39.5%) | **<0.001** |
| Comparison to Q1 |  |  | 0.266 | 0.141 | 0.255 | **0.051** |  |
| Comparison to Q2 |  |  |  | 0.684 | 0.991 | 0.416 |  |
| Comparison to Q3 |  |  |  |  | 0.690 | 0.711 |  |
| Comparison to Q4 |  |  |  |  |  | 0.418 |  |
| **peak V̇O_2_ ml·kg^-1^·min^-1^ (mean,SD)** | 19 | 14.5,3.69 | 15.1,3.64 | 16.3,3.99 | 15.8,3.75 | 16.0,3.80 | **<0.001** |
| Comparison to Q1* |  |  | 0.156 | **<0.001** | **<0.001** | **<0.001** |  |
| Comparison to Q2* |  |  |  | **<0.001** | **0.040** | **0.006** |  |
| Comparison to Q3* |  |  |  |  | 0.582 | 0.930 |  |
| Comparison to Q4* |  |  |  |  |  | 0.962 |  |
| **VE/V̇CO_2_ (mean, SD)** | 27 | 36.8,7.36 | 35.9,6.64 | 35.3,6.46 | 35.9,6.60 | 35.3,7.10 | **0.004** |
| Comparison to Q1* |  |  | 0.188 | **0.018** | 0.261 | **0.009** |  |
| Comparison to Q2* |  |  |  | 0.846 | 1.00 | 0.803 |  |
| Comparison to Q3* |  |  |  |  | 0.783 | 1.000 |  |
| Comparison to Q4* |  |  |  |  |  | 0.731 |  |
| **Right Hand Grip (mean,SD)** | 1353 | 31.1,10.01 | 31.8,10.05 | 31.2,9.45 | 30.4,9.84 | 31.4,9.85 | 0.839 |
| Comparison to Q1* |  |  | 0.963 | 1.000 | 0.972 | 0.999 |  |
| Comparison to Q2* |  |  |  | 0.992 | 0.774 | 0.997 |  |
| Comparison to Q3* |  |  |  |  | 0.968 | 1.000 |  |
| Comparison to Q4* |  |  |  |  |  | 0.941 |  |
| **Left Hand Grip (mean, SD)** | 1354 | 28.7,9.69 | 29.8,9.69 | 29.4,9.39 | 28.7,9.55 | 29.6,9.20 | 0.811 |
| Comparison to Q1* |  |  | 0.863 | 0.981 | 1.000 | 0.936 |  |
| Comparison to Q2* |  |  |  | 0.998 | 0.887 | 1.000 |  |
| Comparison to Q3* |  |  |  |  | 0.983 | 1.000 |  |
| Comparison to Q4* |  |  |  |  |  | 0.945 |  |

**Vascular patients only – comparison of CPET measures by IMD quintile**

*CPET variables comparison displayed for each IMD quintile (Q1 – most deprived to Q5 – least deprived). Data is presented as mean, SD, unless otherwise stated. Percentages are representative of the proportion of patients in each IMD quintile with the variable, apart from the first row which is a percentage of the total population. Significant p-values(p<0.05) are in bold. *Tukey-adjusted post-hoc p-values. **Main effect of ANOVA.*

**Multivariable logistic regression model – GET<11 ml·kg^-1^·min^-1^ as independent variable.** Q1 (most deprived) is the reference value for deprivation. Significant p-values (p<0.05) are in bold.

| Predictor | Odds ratio | Lower CI | Upper CI | p-value |
| --- | --- | --- | --- | --- |
| Age | 1.04 | 1.02 | 1.05 | **<0.001** |
| Sex (Female-Male) | 3.65 | 2.67 | 4.99 | **<0.001** |
| BMI | 1.08 | 1.05 | 1.10 | **<0.001** |
| Current Smoker (Yes-No) | 0.94 | 0.72 | 1.24 | 0.676 |
| RCRI (2-1) | 1.75 | 1.38 | 2.22 | **<0.001** |
| RCRI (3-1) | 2.80 | 1.79 | 4.37 | **<0.001** |
| RCRI (4-1) | 8.31 | 2.75 | 25.18 | **<0.001** |
| FEV_1_/FVC | 0.98 | 0.97 | 0.99 | **<0.001** |
| IMD quintile (2-1) | 0.86 | 0.62 | 1.19 | 0.361 |
| IMD quintile (3-1) | 0.75 | 0.53 | 1.07 | 0.116 |
| IMD quintile (4-1) | 0.80 | 0.58 | 1.12 | 0.193 |
| IMD quintile (5-1) | 0.73 | 0.52 | 1.03 | 0.076 |

**Multivariable linear regression model – GET as continuous independent variable.** Q1 (most deprived) is the reference value for deprivation. Significant p-values (p<0.05) are in bold.

| Predictor | Estimate (β) | Lower CI | Upper CI | p-value |
| --- | --- | --- | --- | --- |
| Age | -0.03 | -0.05 | -0.02 | **<0.001** |
| Sex (Female-Male) | -1.61 | -1.90 | -1.32 | **<0.001** |
| BMI | -0.08 | -0.11 | -0.06 | **<0.001** |
| Current Smoker (Yes-No) | -0.04 | -0.29 | 0.22 | 0.787 |
| RCRI (2-1) | -0.75 | -0.98 | -0.52 | **<0.001** |
| RCRI (3-1) | -1.31 | -1.74 | -0.88 | **<0.001** |
| RCRI (4-1) | -2.41 | -3.28 | -1.54 | **<0.001** |
| FEV_1_/FVC | 0.03 | 0.02 | 0.03 | **<0.001** |
| IMD quintile (2-1) | 0.23 | -0.09 | 0.54 | 0.154 |
| IMD quintile (3-1) | 0.60 | 0.26 | 0.93 | **<0.001** |
| IMD quintile (4-1) | 0.31 | -0.01 | 0.62 | 0.055 |
| IMD quintile (5-1) | 0.38 | 0.05 | 0.70 | **0.023** |

**Multivariable linear regression model – Peak V̇O2 as independent variable.** Q1 (most deprived) is the reference value for deprivation. Significant p-values (p<0.05) are in bold.

| Predictor | Estimate (β) | Lower CI | Upper CI | p-value |
| --- | --- | --- | --- | --- |
| Age | -0.05 | -0.07 | -0.03 | **<0.001** |
| Sex (Female-Male) | -3.13 | -3.51 | -2.76 | **<0.001** |
| BMI | -0.14 | -0.17 | -0.11 | **<0.001** |
| Current Smoker (Yes-No) | -0.33 | -0.69 | 0.03 | 0.069 |
| RCRI (2-1) | -1.22 | -1.55 | -0.89 | **<0.001** |
| RCRI (3-1) | -2.27 | -2.84 | -1.70 | **<0.001** |
| RCRI (4-1) | -4.20 | -5.25 | -3.14 | **<0.001** |
| FEV_1_/FVC ratio | 0.06 | 0.04 | 0.07 | **<0.001** |
| IMD quintile (2-1) | 0.36 | -0.08 | 0.79 | 0.106 |
| IMD quintile (3-1) | 1.52 | 1.04 | 1.99 | **<0.001** |
| IMD quintile (4-1) | 1.14 | 0.69 | 1.58 | **<0.001** |
| IMD quintile (5-1) | 1.14 | 0.68 | 1.61 | **<0.001** |

**Multivariable linear regression model – VE/V̇CO_2_ as independent variable.** Q1 (most deprived) is the reference value for deprivation. Significant p-values (p<0.05) are in bold.

| Predictor | Estimate (β) | Lower CI | Upper CI | p-value |
| --- | --- | --- | --- | --- |
| Age | 0.16 | 0.12 | 0.19 | **<0.001** |
| Sex (Female-Male) | 1.20 | 0.49 | 1.90 | **<0.001** |
| BMI | -0.29 | -0.35 | -0.23 | **<0.001** |
| Current Smoker (Yes-No) | 1.44 | 0.78 | 2.10 | **<0.001** |
| RCRI (2-1) | 1.29 | 0.68 | 1.90 | **<0.001** |
| RCRI (3-1) | 2.31 | 1.26 | 3.37 | **<0.001** |
| RCRI (4-1) | 4.49 | 2.44 | 6.54 | **<0.001** |
| FEV_1_/FVC | -0.12 | -0.14 | -0.10 | **<0.001** |
| IMD quintile (2-1) | -0.76 | -1.56 | 0.04 | 0.063 |
| IMD quintile (3-1) | -1.61 | -2.50 | -0.72 | **<0.001** |
| IMD quintile (4-1) | -0.82 | -1.64 | 0.00 | 0.051 |
| IMD quintile (5-1) | -1.42 | -2.28 | -0.56 | **0.001** |
